# The rising role of cognitive reserve and associated compensatory brain networks in spinocerebellar ataxia type 2

**DOI:** 10.1007/s00415-023-11855-3

**Published:** 2023-07-08

**Authors:** Libera Siciliano, Giusy Olivito, Nicole Urbini, Maria Caterina Silveri, Maria Leggio

**Affiliations:** 1 Department of Psychology, Sapienza University of Rome, 00185 Rome, Italy; 2 Ataxia Laboratory, Fondazione Santa Lucia IRCCS, 00179 Rome, Italy; 3 Department of Psychology, Catholic University of the Sacred Heart, 20123 Milan, Italy

**Keywords:** Cerebellum, Compensation mechanisms, SCA2, Cognitive functioning, NBS, Functional connectivity

## Abstract

Pre-existing or enhanced cognitive abilities influence symptom onset and severity in neurodegenerative diseases, which improve an individual’s ability to deal with neurodegeneration. This process is named cognitive reserve (CR), and it has acquired high visibility in the field of neurodegeneration. However, the investigation of CR has been neglected in the context of cerebellar neurodegenerative disorders. The present study assessed CR and its impact on cognitive abilities in spinocerebellar ataxia type 2 (SCA2), which is a rare cerebellar neurodegenerative disease. We investigated the existence of CR networks in terms of compensatory mechanisms and neural reserve driven by increased cerebello-cerebral functional connectivity. The CR of 12 SCA2 patients was assessed using the Cognitive Reserve Index Questionnaire (CRIq), which was developed for appraising life-span CR. Patients underwent several neuropsychological tests to evaluate cognitive functioning and a functional MRI examination. Network based statistics analysis was used to assess functional brain networks. The results revealed significant correlations of CRIq measures with cognitive domains and patterns of increased connectivity in specific cerebellar and cerebral regions, which likely indicated CR networks. This study showed that CR may influence disease-related cognitive deficits, and it was related to the effective use of specific cerebello-cerebral networks that reflect a CR biomarker.

**Supplementary Information:**

The online version contains supplementary material available at 10.1007/s00415-023-11855-3.

## Introduction

Early assumptions about cognitive reserve (CR) trace back to the reserve hypothesis introduced by Stern and colleagues almost 30 years ago [1, 2]. The hypothesis posits that the exposure to plentiful and diverse lifetime experiences may act as a protective factor encouraging brain enrichment and resilient coping to neurological damage [1, 2]. Distinct conceptualizations of reserves have been proposed. Brain reserve refers to brain resilience driven by structural residual intact neurons/dendrites/synapses after damage [2, 3]. Cognitive reserve relates to cognitive functions that have been efficiently reinforced during life and promote effective handling of brain damage-related decline [2, 3]. Several factors, activities and daily life habits account for reserve enhancement and have been used as indirect proxies of CR, such as education and work-related achievement, physical (walking, running and cycling), cognitive (reading, writing and painting) and social (volunteering and joining group leisure activities) activities, as well as healthy routines (healthy drinking and nutrition and non-smoking) [2, 3].

CR is an active process. Assuming an equal amount of brain damage, some people achieve better functioning than others due to the efficacy or capacity of cognitive processes [4]. Different types of neural operations may support CR [2]. Neural reserve (NR) refers to the efficacy of certain brain regions or networks in making themselves less susceptible to neurodegenerative processes [2, 5]. The NR mechanisms support information processing despite brain deterioration [2, 5]. Neural compensation (NC) refers to the assumption that individuals with brain damage may recruit networks to accomplish a given function that are not usually activated for these functions [2]. The mechanism supported by NC is aimed to compensate for regions impaired by the disease [2]. NC has also been described as the capacity of structurally atrophic cerebral areas to remain functional despite neurodegeneration [6–9]. This process is expressed in terms of increased functional connectivity (FC) [6–9]. All of these processes may be involved when individuals deal with brain diseases [10].

Resting-state fMRI (rsfMRI) has been used as a powerful means to estimate intrinsic neural activity among or within brain networks associated with CR in patients with brain degeneration of different etiologies. rsfMRI studies on patients with Alzheimer’s disease found that higher CR positively correlated with the FC of the left frontal lobe [11], the right middle temporal pole [12] and posterior cingulate gyrus [13] and greater network efficiency in the frontal region [14] and right middle temporal pole [12]. Higher CR based on education level was associated with higher intrinsic brain activity in the PHG and the inferior parietal lobe (IPL) in patients with mild cognitive impairment (MCI), and the PHG was also associated with the maintenance of executive function sed intrinsic activity in the aforementioned brain regions likely reflected the recruitment of compensatory resources to counterbalance the effect of the disease [15]. Enhanced connectivity in the DMN and networks involving cortical and cerebellar regions has been reported in patients with amyotrophic lateral sclerosis [16, 17]. These studies suggest that increased patterns of connectivity delay the occurrence of symptoms and compensate for the impacts of neurodegeneration [16, 17].

SCA2 is a rare autosomal dominant inherited cerebellar neurodegenerative disease that is characterized by a progressive cerebellar syndrome with primary clinical manifestations involving motor control and coordination [18]. As the disease advances, the SCA2 phenotype is also associated with the manifestation of cognitive, emotional and social deficits, which are consistent with the description of the well-known cerebellar cognitive-affective syndrome [19–23]. SCA2 brain degeneration consists of brainstem and cerebellar damage in the early stages of the disease and of cerebral cortical atrophy as the disease advances. Patterns of reduced FC in cerebro-cerebellar networks have been consistently reported in patients affected by SCA2 and linked with the core neurodegenerative process affecting definite cerebellar zones [22, 23]. Enhanced FC within cortical areas of the DMN and the frontoparietal networks and between the cerebellum and areas in the parietal lobe has been reported in patients affected by SCA2 [9]. The higher FC in the aforementioned networks correlated with better performance in tasks evaluating motor, learning, and attentional domains [9]. The authors suggested that these patterns of increased FC reflected compensation and allowed structurally atrophic cerebellar areas to remain functional despite neurodegeneration [9]. Proof of the existence of brain network reorganization aimed at compensating for functional outcomes in patients with SCA2 comes from a recent study from our research group on motor reserve and subtending networks in this population of patients [24]. Notably, higher motor reserve was associated with a lower severity of motor symptoms and better performance in executive function in SCA2 [24]. The study revealed patterns of increased FC in cerebral and cerebellar nodes involved in motor functions that correlated with life-span motor reserve. These results suggested the presence of motor reserve networks supporting compensatory mechanisms that allowed for coping with the disease [24].

The concept of cerebellar reserve must also be mentioned. Due to its wide plastic reorganization, the cerebellum exhibits experience-related sensitivity [25]. As a result, the cerebellum compensates and repairs functionality in response to focal and degenerative cerebellar alterations. This process is referred to as the cerebellar reserve [25]. The cytoarchitectonic and functional arrangement of the cerebellum favor the hypothesis that cerebellar modulatory activity on cortical and subcortical projection areas induce brain network rearrangement and functional compensation in the presence of degeneration [25].

Despite these premises, thus far no study investigated cognitive reserve and associated neural networks in cerebellar neurodegenerative disease. To overcome this gap, the present study examined life-span CR using the Cognitive Reserve Index questionnaire (CRIq) [26] in patients affected by SCA2 and the existing correlations between CRIq and cognitive performance. This study examined the presence of compensatory neural mechanisms in SCA2 expressed as increased FC and the correlations between these mechanisms and the CRIq. Specifically, we investigated the patterns of increased cerebellar and cerebral internodal connectivity using network-based statistics (NBS). This technique allows us to depict the brain as a graph described in terms of networks constituted by specific regions named nodes, which are functionally connected by edges [27, 28].

## Methods

### Participants

**Fig. 1:**
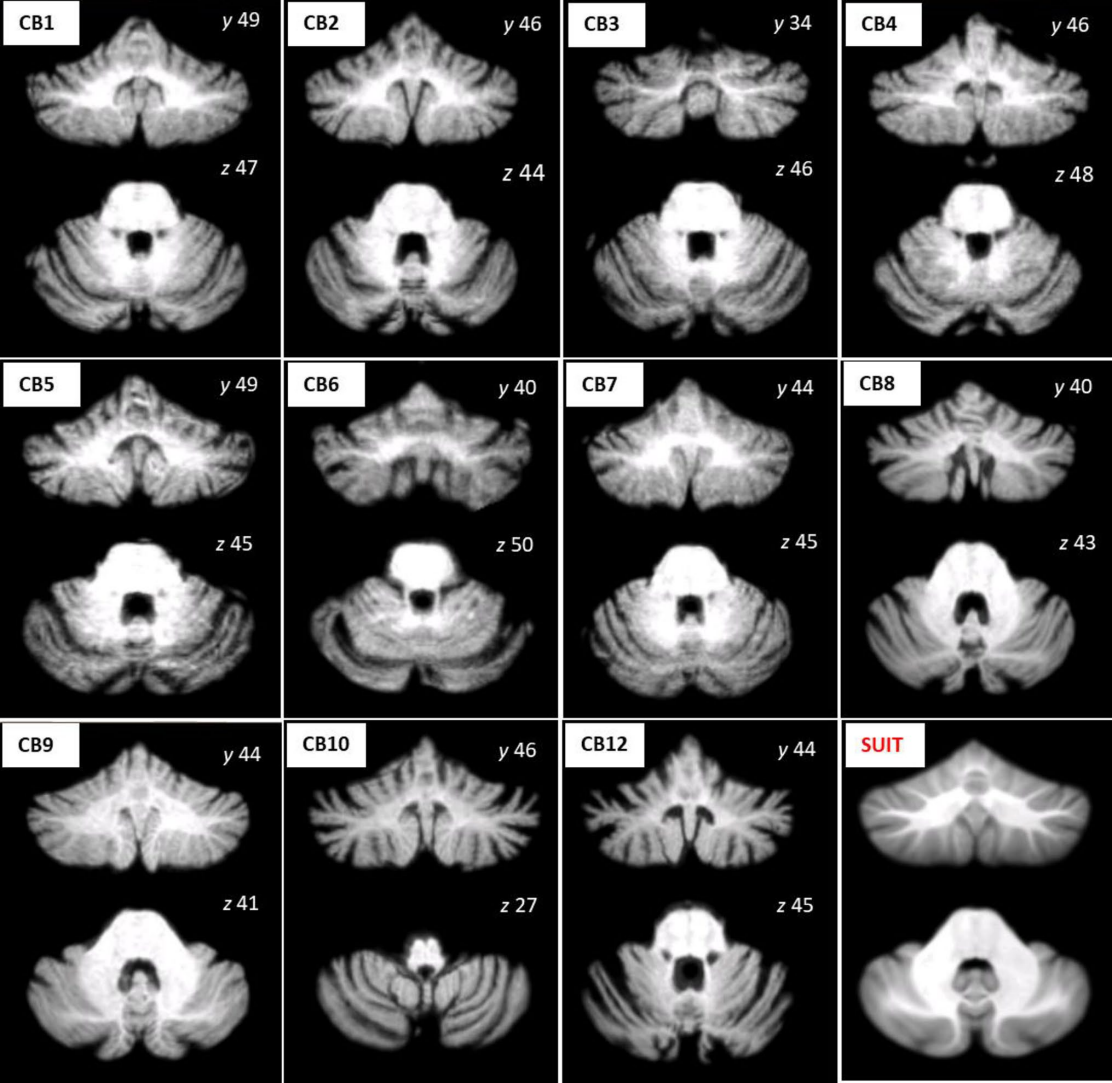
T1-weighted images of each SCA2 patient normalized to the Spatially Unbiased atlas template of the cerebellum and brainstem (SUIT) (Diedrichsen et al., 2006, in the lower panel to the left) are reported in representative sections showing the spectrum of cerebellar atrophy

The present study enrolled 12 patients with a genetic diagnosis of SCA2 [female/male: 7/5; mean age/SD at the time of the clinical and magnetic resonance imaging (MRI) assessment: 48.3/8.3 (years); mean educational level/SD: 14.4/3.7 (years)] from the Ataxia Laboratory of the IRCCS Santa Lucia Foundation. All patients received their diagnosis at least 6 months prior and showed no other neurological signs with the exception of CB4, who presented the Babinski sign. An expert neurologist examined the patients to confirm the presence of pure cerebellar motor symptoms and assessed the severity of their ataxia-related motor symptoms using the International Cooperative Ataxia Rating Scale (ICARS) [29]. For a detailed report of ICARS scores of each SCA2 patient, see Table S1 in the Supplementary Materials. The demographic and clinical characteristics of the patients are reported in Table 1. All patients underwent MRI examination except CB11, who had a coronary stent that was incompatible with the MRI scanner. Conventional MRI scans were inspected by an expert neuroradiologist confirming the absence of macroscopic extra-cerebellar brain abnormalities and revealing the presence of diffuse cerebellar gray matter atrophy as showed in Fig. 1. The patients in the present study correspond to a previous study that investigated motor reserve in SCA2 [24] and partially overlapped with the samples of other studies [22, 23, 30].

**Table 1:** Demographic and clinical characteristics of SCA2 patients

ID	Age	M/F	Education (years)	Disease duration (months)	CAG	ICARS
CB1	42	F	13	7	35 ± 1	47
CB2	40	F	18	147	47 ± 1	26
CB3	64	M	17	42	35 ± 1	28
CB4	54	F	18	45	37 ± 1	27
CB5	60	F	8	42	37	31
CB6	43	F	13	154	n.a	28
CB7	38	F	13	114	42	39
CB8	42	M	18	64	39	17
CB9	54	M	18	n.a	n.a	24
CB10	48	M	13	47	38 ± 1	29
CB11	51	M	8	156	37	24
CB12	44	F	16	118	n.a	61
Means (SD)	48.33 (8.29)	5/7	14.42 (3.70)	85.09 (53.71)	–	31.75 (11.91)

*F* female; *M* male; *CAG* Number of expanded triplets. *N.a*. information about CAG size not present for patients CB29, CB40, CB49 since, at the time of diagnosis, genetic testing did not include triplet repeat number determination; *ICARS* International Cooperative Ataxia Rating Scale. ICARS range: minimum score 0 (absence of motor deficits), maximum score 100 (maximum presence of motor deficits) [29]. Disease duration corresponds to the time period from the genetic testing

Regarding MRI analysis, we used a control group composed of 24 healthy subjects (HS) with no history of neurological or psychiatric illness (female/male: 11/13; mean age/SD: 41.5/13.6; mean education level/SD: 15.5/2.9) based on retrospective MRI data collected from 2014 to 2019 at the Neuroimaging Laboratory of the IRCCS Santa Lucia Foundation. The sample size was consistent with a previous study that compared MRI data between SCA2 patients and healthy subjects using NBS analysis [22]. A *t* test revealed no significant differences between SCA2 patients and HS in age (*t* = −1,47; *p* = 0.151) or educational level (*t* = 0.45, *p* = 0.66). The Ethics Committee of the Santa Lucia Foundation approved this study, which was performed in accordance with the principles expressed in the Declaration of Helsinki (approval code: CE/PROG.932). Written informed consent was obtained from each subject.

### Cognitive reserve

CR was measured using the CRIq [26], which is an internationally validated questionnaire specifically developed to assess acquired cognitive reserve over a lifetime, from the age of 18 years. Expert health professionals administered the questionnaire using semi-structured interviews that allowed us to estimate the CR of the patients from the age of 18 years to the time of cognitive and MRI evaluations. The CRIq is comprised of 20 items divided into three main subscores: CRI-Education (CRIq_Edu), which considers the years of education and additional years of training courses attended for at least 6 months; CRI-Working Activity (CRIq_WA), an estimate of the individual’s years of work in adulthood with five different levels ranging from lower scores for primarily manual work that do not require particular skills to higher scores for highly responsible and intellectual occupations; and CRI-LeisureTime (CRIq_LA), reflecting the leisure time spent in cognitively stimulating activities (reading, traveling, housekeeping and artistic activities). The three subscores and the total index of CR (CRI_Tot) were calculated using an Excel file for automatic calculation, which was made accessible by the authors who designed the questionnaire (available at http://cri.psy.unipd.it). Each CRIq subscale and the CRIq_Tot are expressed on a scale with mean = 100 and standard deviation = 15. For CRIq_Tot, a result ≤ 70 was classified as low, and a result ≥ 130 was classified as high [26]. Therefore, the presence of a great CR corresponded to higher scores in the CRIq subscales and total index.

### Cognitive measures

Each patient underwent a comprehensive battery of neuropsychological tests to evaluate several cognitive domains:

- Intellectual functioning: Wechsler Adult Intelligence Scale—Revised [31, 32];
- Short-term memory: Immediate recall of Rey’s 15 words [33], forward and backward Digit Span [34, 35], Corsi Test [36], immediate recall of Short-Story Recall Task [37];
- Long-term memory: Delayed recall of Rey’s 15 words [33], Delayed recall of Short-Story Recall task[37];
- Attention: Multiple Features Target Cancellation Task (MFTC)—accuracy scores [38], Line Cancellation Task—accuracy scores [39];
- Executive functions: Phonological fluency [40], Wisconsin Card Sorting Test—perseverative errors [41], Stroop Test—accuracy score [42, 43];
- Processing speed: Stroop Test—execution time [42, 43], Multiple Features Target Cancellation task (MFTC)—execution time [38], Line Cancellation Task—execution time [39].

### MRI data protocol

All participants underwent an MRI inspection at 3 T (Magnetom Allegra, Siemens, Erlangen, Germany) that included the following acquisitions: (1) dual-echo turbo spin-echo (TSE) (TR = 6190 ms, TE = 12/109 ms); (2) fast-FLAIR (TR = 8170 ms, 204TE = 96 ms, TI = 2100 ms); (3) 3D modified driven equilibrium Fourier transform (MDEFT) scan (TR = 1338 ms, TE = 2.4 ms, matrix = 256 × 224 × 176, in-plane FOV = 250 × 250 mm^2^, slice thickness = 1 mm); (4) T2*-weighted echo-planar imaging (EPI) sensitized to blood-oxygenation-level-dependent imaging (BOLD) contrast (TR: 2080 ms, TE: 30 ms, 32 axial slices parallel to AC-PC line, matrix: 64 × 64, pixel size: 3 × 3 mm^2^, slice thickness: 2.5 mm, flip angle: 70°) for resting state fMRI. During rest, BOLD echo-planar images were acquired for 7 min and 20 s periods, with a total of 220 volumes. During this acquisition, patients were asked not to think about anything in particular, to keep their eyes closed, and not to fall asleep. A practiced neuroradiologist acquired and revised the TSE scans of all patients to inspect the anatomy of the brain and verify the presence of macroscopic structural alterations of extracerebellar areas. According to the inclusion criteria, conventional MRI scans of the control subjects were inspected to exclude any pathological conditions.

#### Resting-state fMRI data pre-processing

The data were pre-processed via statistical parametric mapping [Wellcome Department of Imaging Neuroscience; SPM8 (http://www.fil.ion.ucl.ac.uk/spm/)] and in-house software implemented in MATLAB (MathWorks Inc., Natick, MA, USA). The first four volumes of the fMRI series for each subject were rejected to permit T1 equilibration effects. The data pre-processing consisted of the following procedures: correction for head motion, compensation for slice-dependent time shifts, normalization to the EPI template in MNI coordinates supplied with SPM8, and smoothing with a 3D Gaussian kernel with 8-mm^3^ full width at half-maximum. The parameters of motion assessed during correction were inspected for every dataset to guarantee that the maximum absolute shift did not exceed 2 mm, and the maximum absolute rotation did not exceed 1.5°. The global temporal drift was removed using a third-order polynomial fit, and the signal was regressed against the realignment parameters and balanced over whole-brain voxels to eliminate further possible causes of bias. All images were filtered using a phase-insensitive bandpass filter (pass band 0.01–0.08 Hz) to reduce the consequences of low-frequency drift and high-frequency physiological noise.

#### Network-based statistics

A body of 116 nodes determined by the Automated Anatomical Labeling (AAL) Atlas was first defined to obtain a connectivity matrix for every subject. The mean time course of every node was estimated as the average of the fMRI time series from all voxels in a certain region. We obtained correlation matrices by estimating the correlation between all mean signals in the pairs of nodes, as detailed by Serra et al. [44]. Therefore, changes in FC between definite cerebellar and cerebral “nodes” were detected. The statistical comparison was performed using the NBS tool developed by Zalensky et al. [45]. The comparison of FC matrices between patients and controls was performed using a two-sample *t* test, with 5000 permutations and the statistical significance (*p* value) set at 0.05, adjusted for multiple comparisons using NBS correction [45].

### Statistical analyses

First, we clustered the different cognitive tests by grouping them together for each investigated cognitive function. The raw scores of each subject in the various tests were converted into *z* scores. As reference means, normative data were used for the following tests: Rey’s 15 Words, Short Story Test, MFTC, and the Line Cancellation Task. The population mean scores used as a reference for the other tests were obtained from the scores of specific healthy control groups in our databases who did not differ significantly from patients in mean age and education (*t* test—considered significant for *p* < 0.05). A composite *z* score was calculated for each cognitive domain by calculating the mean *z* scores of the tests belonging to that cognitive cluster. The resulting *z* scores for each cognitive domain are reported in Fig. S1 in the Supplementary Materials. The demographic characteristics and the data of the cognitive tests of the control groups are reported in Table S2. Neuropsychological data of SCA2 patients are reported in Table S3.

Statistical analyses were performed using the Statistical Package for the Social Sciences (SPSS) version 27. We investigated the relationship between CRIq subscores and (i) the patients’ performance in each cognitive domain and (ii) the patterns of increased internodal FC. We tested the assumption of normality using the Kolmogorov–Smirnov and Shapiro–Wilk tests. Because normality was not assumed, we used Spearman’s rank order correlation test. To confirm the use of this coefficient, we inspected scatterplots of each pair of variable associations to verify the presence of a monotonic relationship. To avoid Type I error, the Bonferroni correction was used to correct for multiple testing.

## Results

The CRIq scores of each SCA2 patient are listed in Table 2.

**Table 2:** Individual results of Cognitive Reserve Index questionnaire (CRIq) in SCA2 patients

ID	CRIq_Edu	CRIq_WA	CRIq_LA	CRIq_Tot
CB1	96	94	92	92
CB2	114	91	111	107
CB3	120	117	117	124
CB4	137	132	132	145
CB5	90	91	113	97
CB6	109	83	104	98
CB7	99	100	95	97
CB8	112	106	120	116
CB9	118	115	97	113
CB10	105	118	121	120
CB11	93	98	90	91
CB12	106	101	95	101
Means (SD)	108.25 (13.23)	103.83 (14.19)	107.25 (13.66)	108.42 (15.93)

Corrected CRIq scores in each subscale and the total index of cognitive reserve are reported. *CRIq_Edu* CRIq-Education; *CRIq_WA *CRIq-WorkingActivity; *CRIq_LA *CRIq-LeisureTime; *CRIq_Tot* CRIq-Total Index. Each CRIq subscale and the CRIq_Tot are expressed on a scale with mean = 100 and standard deviation = 15. For CRIq_Tot a result ≤ 70 is classified as low and a result ≥ 130 is classified as high [26]

### Correlation between CRIq scores and cognitive measures

**Fig. 2:**
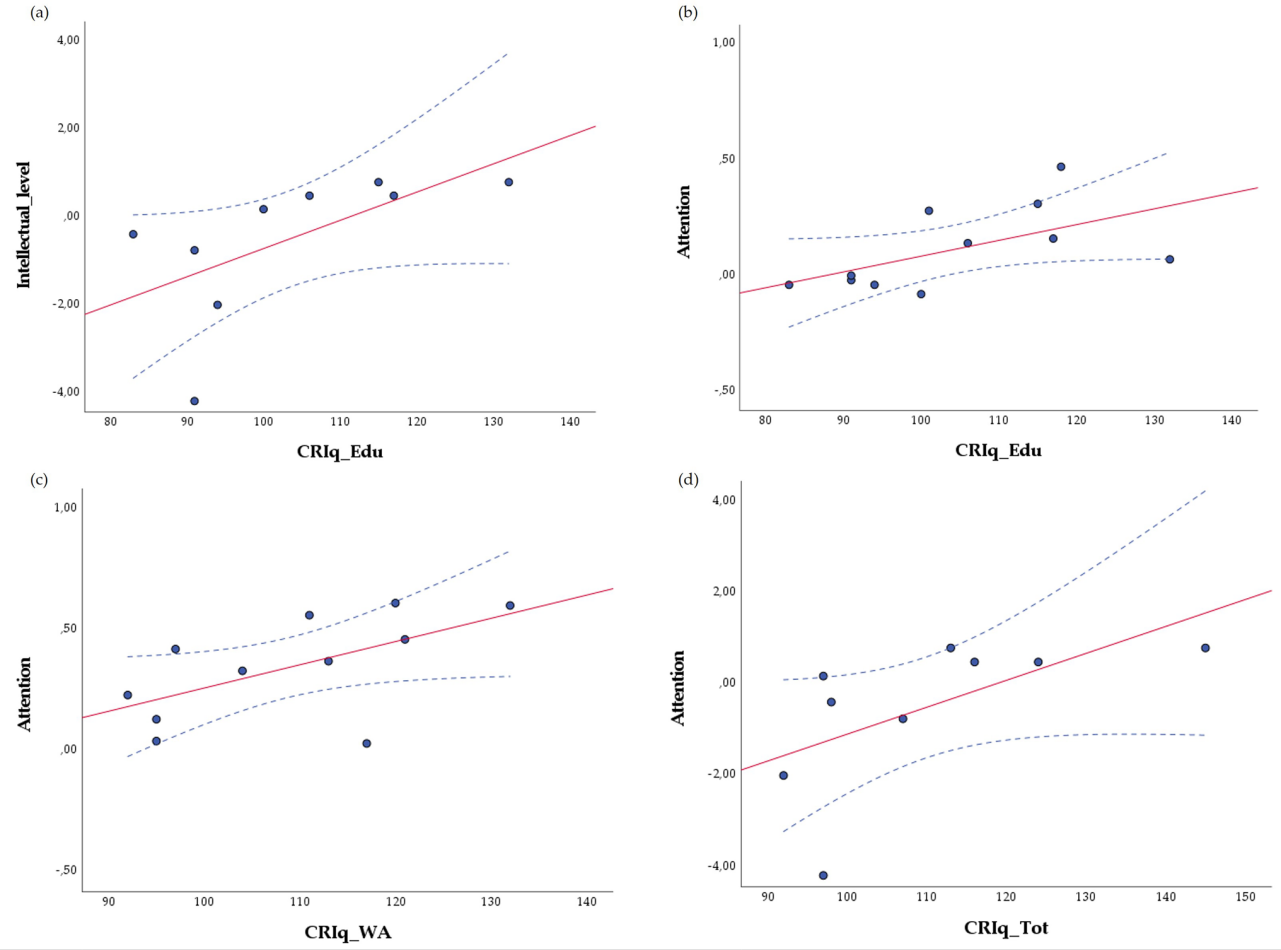
Data scatterplots of significant correlations between CRIq scores and Cognitive Measures: **a** correlation between CRIq_Edu and Intellectual Functioning; **b** correlation between CRIq_Tot and Attention; **c** correlation between CRIq_WA and Attention; **d** correlation between CRIq_Edu and Attention. *CRIq_Edu* CRIq-Education; *CRIq_WA* CRIq-WorkingActivity; *CRIq_LA* CRIq-LeisureTime; *CRIq_Tot* CRIq-Total Index. Dotted lines indicate the 95% confidence intervals

Correlational analysis was performed using Spearman’s coefficient and revealed considerable correlations between different CRIq subscales and some measures of cognitive domains (see Fig. S1 in the Supplementary Materials for patients’ cognitive domains expressed in *z* scores). Positive correlations were detected between Attention and CRIq_Edu (*r* = 0.832; *p* = 0.005), CRIq_WA (*r* = 0.827; *p* = 0.006) and CRIq_Tot (*r* = 0.810; *p* = 0.008), which revealed a relationship between attention abilities and the CR resulting from education and cognitively demanding working activities. A significant positive correlation was evidenced between CRIq_Edu and Intellectual Level (*r* = 0.635; *p* = 0.026), which demonstrated that a higher CR developed via education corresponded to a greater individual intellectual level. The results of the Spearman’s rank order correlation analyses are reported in Table 3. Data scatterplots were generated for significant correlations and are shown in Fig. 2. After application of the Bonferroni correction for the family-wise error rate, the correlations between CRIq scores and the cognitive domains were no longer statistically significant (see “Discussion” section).

**Table 3:** Correlations between Cognitive Reserve Index Questionnaire (CRIq) scores and cognitive measures

	Intellectual functioning	Short-term memory	Long-term memory	Attention	Executive functions	Processing speed
CRIq-Edu	***r* = *0.635***	*r* = 0.322	*r* = 0.238	***r* = *0.832***	*r* = − 0.231	*r* = 0.133
	***p* = *0.026***	*p* = 0.308	*p* = 0.457	***p* = *0.005***	*p* = 0.471	*p* = 0.681
CRIq-WA	*r* = 0.244	*r* = 0.175	*r* = − 0.144	***r* = *0.827***	*r* = − 0.109	*r* = 0.046
	*p* = 0.444	*p* = 0.586	*p* = 0.656	***p* = *0.006***	*p* = 0.737	*p* = 0.888
CRIq-LA	*r* = 0.409	*r* = 0.518	*r* = 0.504	*r* = 0.395	*r* = 0.277	*r* = − 0.263
	*p* = 0.186	*p* = 0.084	*p* = 0.094	*p* = 0.293	*p* = 0.384	*p* = 0.409
CRIq-Tot	*r* = 0.515	*r* = 0.392	*r* = 0.228	***r* = *0.810***	*r* = − 0.014	*r* = − 0.042
	*p* = 0.087	*p* = 0.207	*p* = 0.477	***p* = *0.008***	*p* = 0.966	*p* = 0.897

Significant correlations (p ≤ 0.05) are presented in bold italic type. *CRIq_Edu* CRIq-Education; *CRIq_WA* CRIq-WorkingActivity; *CRIq_LA *CRIq-LeisureTime; *CRIq_Tot* CRIq-Total Index

### Functional connectivity results and correlation with CRIq

NBS analysis revealed increased internodal connectivity in SCA2 patients compared to controls, which overall involved 16 nodes and 12 edges. Specifically, increased FC was detected in purely motor areas [e.g., bilateral supplementary motor area (SMA), right medial frontal cortex (MFC) and left cerebellar lobules IV-V, as discussed in Siciliano et al. [24] and primarily cognitive areas (e.g., left parahippocampal gyrus, bilateral fusiform cortex and cerebellar vermis X). More detailed results of the NBS analysis are provided in Table 4, which shows the pairwise cerebellar and cerebral nodes of significant FC increases in SCA2 patients.

**Table 4:** Edges of significant FC increases in SCA2 patients

Pairwise brain regions		*t*-Values
*Cerebro-Cerebral Nodes*		
R MFC	R SMA	4.28
	L SMA	4.50
	R superior temporal pole	4.26
L Cuneus	L inferior parietal lobule	4.53
*Cerebellar-Cerebral nodes*		
Vermis I–II	R Fusiform Cortex	4.09
	L Parahippocampal Gyrus	4.04
Vermis IV–V	R Occipital Medial Cortex	4.00
Vermis X	R Fusiform Cortex	4.39
	L Fusiform Cortex	4.44
	L Parahippocampal Gyrus	3.95
R Lobule X	L Medial Temporal Pole	3.82
*Cerebello-Cerebellar Nodes*		
Vermis X	L Lobules IV–V	3.64

Patterns of increased functional connectivity into pairwise cerebellar and cerebral regions in SCA2 patients compared with controls (*p* value < 0.05 after FDR correction using network-based statistics) *t-Values are reported; *R* right; *L* left; *MFC* medial frontal cortex; *SMA*  supplementary motor area.

**Fig. 3:**
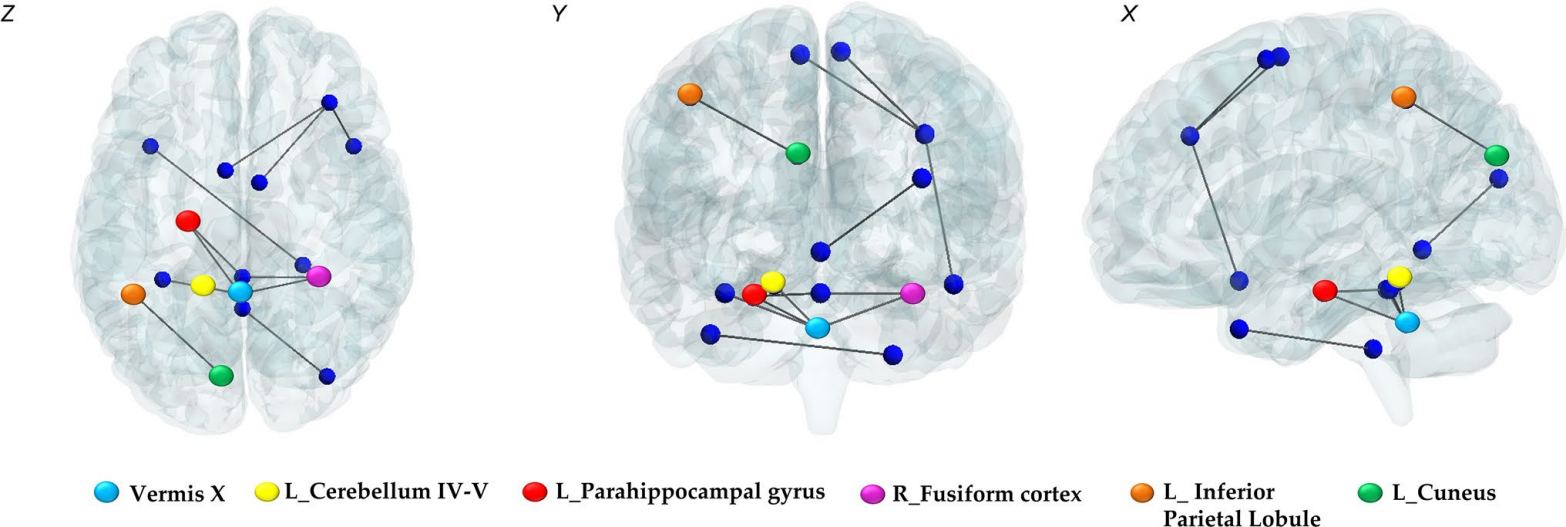
Whole-brain functional connectivity: patterns of significantly increased internodal FC in SCA2 patients compared to controls as assessed by means of NBS analysis (FWE = 0.05) (both blue and colored nodes). The nodes marked in different colors are labeled at the bottom of the figure and refer to cerebellar and cortical areas that revealed significant correlations with CRIq in SCA2 patients (see Table 4) BrainNet Viewer (https://www.nitrc.org/projects/bnv/) [46] was used to visualize the brain network in axial (*z*), coronal (*y*) and sagittal (*x*) sections. *L* left; *R*  right

**Fig. 4:**
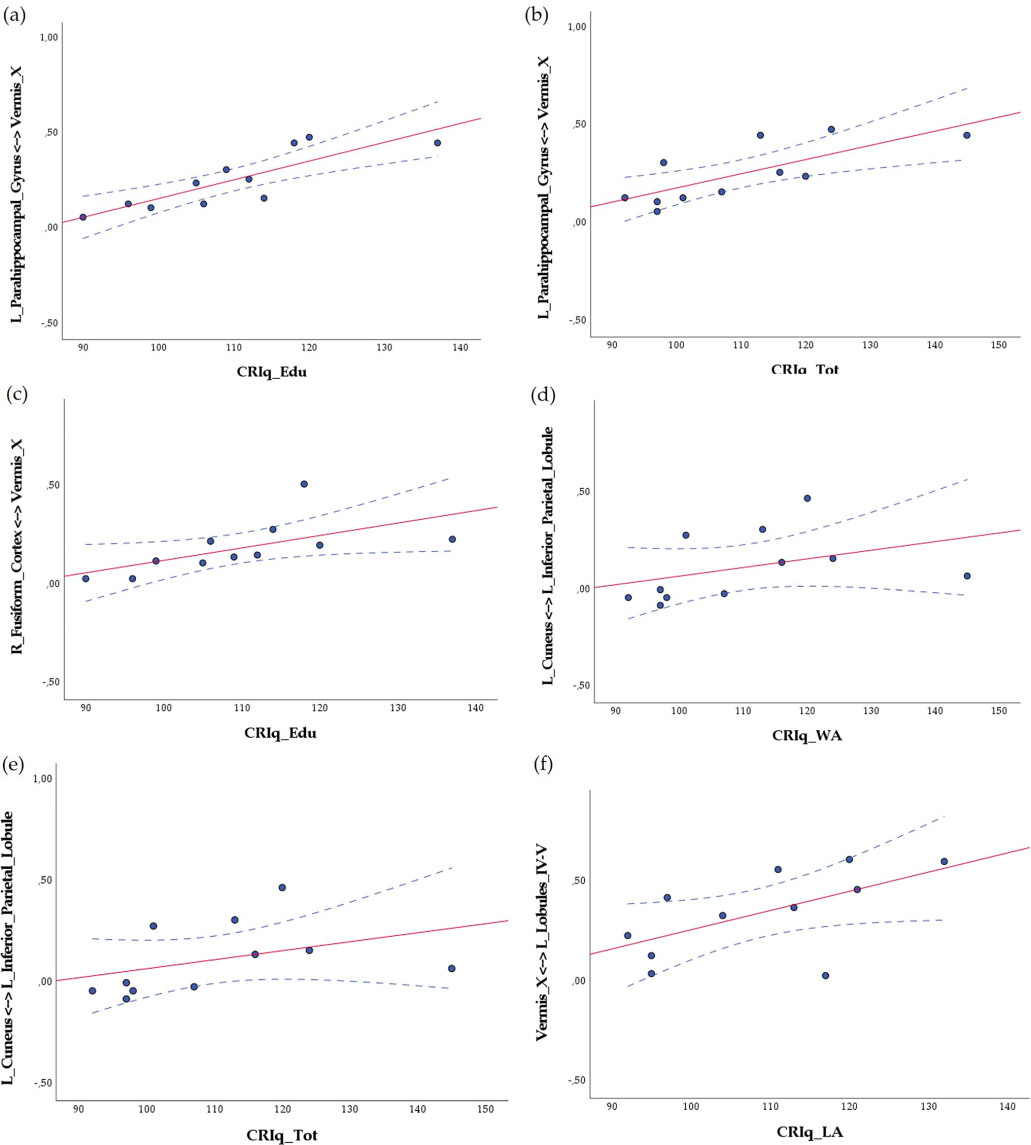
Data scatterplots of significant correlations between CRIq and increased internodal FC between: **a**, **b** the left Parahippocampal Gyrus and cerebellar Vermis X; **c** the right Fusiform Cortex and cerebellar Vermis X; **d**, **e** the left Cuneus and the left Inferior Parietal Lobule; **f** cerebellar Vermis X and left anterior cerebellar Lobules IV–V. The blue dots indicate the values for an individual data point; the red fit lines represent the trend of the data. *CRIq_Edu* CRIq-Education; *CRIq_WA* CRIq-WorkingActivity; *CRIq_LA* CRIq-LeisureTime; *CRIq_Tot* CRIq-Total Index; *R*  right; *L* left. Dotted lines indicate the 95% confidence intervals

Statistical analysis of the CR scores and patterns of increased internodal FC revealed interesting significant correlations with different subscales and cerebral and cerebellar cognitive areas. Strong positive correlations were found between CRIq_Edu (*r* = 0.881; *p* < 0.001) and CRIq_Tot (*r* = 0.783; *p* = 0.004) and increased internodal FC between the left parahippocampal gyrus and cerebellar vermis X. CRIq_Edu also correlated with increased FC between the right fusiform cortex and cerebellar vermis X (*r* = 0.843; *p* = 0.001). A pattern of cerebro-cerebral increased FC between the left cuneus and left inferior parietal lobule positively correlated with CRIq_WA (*r* = 0.685; *p* = 0.020) and CRIq_Tot (*r* = 0.653; *p* = 0.029). A positive correlation was found between CRIq_LA and increased internodal FC within the cerebellum, specifically between vermis X and left lobules IV-V (*r* = 0.606; *p* = 0.048). Cerebellar and cerebral regions that revealed significantly increased FC in SCA2 are shown in Fig. 3. The data scatterplots for significant correlations are reported in Fig. 4. The FC results that significantly correlated with the CRIq scores are reported in Table 5. For a detailed report of all the correlations between patterns of increased internodal FC and CRIq scores, see Table S4 in the Supplementary Materials.

**Table 5:** Patterns of increased internodal FC that significantly correlated with CRIq scores

	CRIq_Edu	CRIq_WA	CRIq_LA	CRIq_Tot
L Parahippocampal Gyrus ↔ Vermis X	***r* = *0.881*** ***p* = *0.000***	*r* = 0.554 *p* = 0.077	*r* = 0.474 *p* = 0.141	***r* = *0.783*** ***p* = *0.004***
L_Cuneus ↔ L Inferior Parietal Lobule	*r* = 0.346 *p* = 0.297	***r* = *0.685*** ***p* = *0.020***	*r* = 0.420 *p* = 0.198	***r* = *0.653*** ***p* = *0.029***
R_Fusiform Cortex ↔ Vermis X	***r* = *0.843*** ***p* = *0.001***	*r* = 0.308 *p* = 0.356	*r* = 0.128 *p* = 0.708	*r* = 0.550 *p* = 0.079
Vermis X ↔ L Lobules IV–V	*r* = 0.300 *p* = 0.370	*r* = 0.205 *p* = 0.545	***r* = *0.606*** ***p* = *0.048***	*r* = 0.419 *p* = 0.199

Significant correlations (*p* ≤ 0.05) are presented in bold italic type. *CRIq_Edu* CRIq-Education; *CRIq_WA* CRIq-WorkingActivity; *CRIq_LA *CRIq-LeisureTime; *CRIq_Tot* CRIq-Total Index; *R *right; *L* left

After correcting for multiple comparisons using Bonferroni correction, the surviving correlations were between CRIq_Edu and the increased FC between cerebellar vermis X and left parahippocampal gyrus and between CRIq_Edu and the increased FC between vermis X and R fusiform cortex.

## Discussion

Cognitive functions in SCA2 patients were underrated until several years ago but have now been widely evaluated, some of whom have been recognized to be deficient in this population [9, 30, 47]. However, cognitive reserve has been largely ignored. Providing proper attention to cognitive dysfunctions in patients affected by a neurodegenerative disorder, such as SCA2, should parallel the attention given to cognitive reserve. Indeed, the undervaluation of both aspects may impact the quality of life of patients and their families. Therefore, boosting CR may benefit cognitive functioning and general coping with the neurodegeneration of patients and family members with the genetic risk of developing the disease. The present study provided the first thorough examination of CR in SCA2 patients and several noteworthy findings on CR and associated rsfMRI connectivity. Overall, our results reported an association between SCA2 patients CR and their cognitive abilities and between CR and patterns of increased internodal FC These results indicated the identification of CR neural substrates.

First, we found that several measures of CR positively correlated with attention performance in SCA2 patients. Specifically, higher attention skills positively correlated with a greater total estimate of CR, greater CR expressed in terms of highly responsible and intellectual working activities and greater CR expressed in terms of years of education. The latter also positively correlated with the intellectual level. Our results are consistent with a recent study that revealed that greater educational attainment and greater occupational position correlated with better performance in attention tasks in patients affected by amnestic MCI (aMCI) and subjects with cognitive decline [48]. Therefore, educational and occupational attainment may denote a CR protective effect on attention functions.

Generally, neurodegenerative progression in SCA2 patients is associated with the occurrence of attentional deficits[47]. A study by our research group on a sample of SCA2 patients found specific impairments in attentional tasks that required higher cognitive demands. Attentional impairments correlated with the degree of gray matter reduction in cerebellar lobules that modulate cognitive processes, such as lobules VII and VIII [47]. Studies on SCA2 patient morphological alterations typically report cerebellar degeneration in anterior (I–II, IV–V) and posterior (VI, VII and VIII) lobules [30, 47, 49, 50]. On the contrary, disease progression spares degeneration in lobule X [49–51]. Patterns of inter-nodal underconnectivity have also been demonstrated in SCA2 patients. Nodes in the posterior cerebellum (i.e., Crus I and Crus II) showed reduced FC with nodes in cortical regions (e.g., superior and middle frontal gyrus), which are related to cognition and emotion [30]. Nodes in the anterior cerebellum (e.g., III, IV, V and vermis IV–V) showed reduced FC with nodes in cortical regions (e.g., precentral and postcentral gyrus) that are related to motor control [30]. Collectively, MRI studies aimed at characterizing morphological and functional alterations in SCA2 patients have not reported degeneration and FC reduction involving cerebellar lobule X. In contrast, this lobule has been implicated in compensatory mechanisms in SCA2 patients [24]. Indeed, patterns of increased inter-nodal FC between lobule X and lobules IV–V correlate with estimates of life-span motor reserve in these patients [24].

The present study found strong positive correlations between greater CR expressed in terms of years of education and increased internodal FC between cerebellar vermis X and the right fusiform cortex and the left PHG. Increased internodal FC between cerebellar vermis X and the left PHG also correlated with the total CR estimate. Increased internodal FC between cerebellar vermis X and cerebellar lobules IV-V correlated with higher CR in terms of the leisure time spent in cognitively stimulating activities (e.g., reading, traveling and artistic activities). Overall, these results are consistent with the hypothesis that CR provides a protective effect that makes patients more capable of using residual or compensatory neural resources to preserve their cognitive abilities. Notably, a recent study conducted with the aim of providing robust and valuable models of normal cerebellar growth across the entire lifespan reported that, contrary to other cerebellar lobule lifespan trajectories, lobules VIIIB and lobule X showed late peak maturation at the age of 30 years and a slow volume increase until 40–50 years old [52]. This cerebellar growth model is similar to hippocampal growth models [53]. These patterns of growth reflect cerebellar involvement in maintained learning and experience-based plasticity [52]. The late peak maturation of these lobules may support the involvement of these two specific cerebellar regions in lifespan adaptation abilities [52]. Our results and the results of Romero and colleagues [52] suggest that lobule X might be involved in neural reserve mechanisms in SCA2 patients. This is consistent with the hypothesis that the efficiency and properties of specific brain regions make these regions less susceptible to neurodegenerative processes [2, 5]. As a consequence, these mechanisms may be responsible for appropriate and optimal information processing despite brain deterioration in other brain regions [2, 5]. Our results are consistent with the concept of cerebellar reserve, which is an outstanding feature of the cerebellar system that reflects self-repair and enables resilient responses to focal lesions and neurodegeneration [25]. Cerebellar reserve is likely related to the ability of the cerebellum to optimize and reorganize diverse functions (e.g., perceptive, motor and cognitive) that allow the detection or adjustment of experience-based internal models [54–57]. These mechanisms are supported by cerebellar plastic reorganization and the presence of diffuse cerebellar-cerebral networks.

Regarding cerebellar plastic reorganization, learning signals induce modifications in different types of cerebellar neurons and consequently cause adjustment of the input‒output organization of the cerebellum [58]. Therefore, cerebellar damage may cause an update of the internal model via synaptic plasticity. Regarding the presence of diffuse cerebellar-cerebral networks, different kinds of information arriving from the cerebral cortex and the periphery converge and integrate within a single cerebellar microzone, which corresponds to a functional unit in the cerebellar cortex [25]. The presence of diffuse and redundant inputs to cerebellar microzones facilitates internal model reorganization within different microzones after damage to one or more microzones that were originally in charge of impaired function [25]. Overall, the updating of internal models may be a key process through which the cerebellar reserve enables resilient motor and cognitive responses despite neurodegeneration. Our results suggest that the cerebellar vermis X is the area in which these mechanisms occur, favoring cognitive reserve in SCA2 patients.

The compensatory recruitment of the PHG as a neuroimaging CR proxy was previously described in MCI patients, and higher CR was associated with the regulation of executive function decline [15].

Together with increased FC involving cerebellar lobules, we also detected patterns of increased FC in cerebro-cerebral nodes involved in cognitive processing. Specifically, increased levels of total CR and CR expressed in terms of highly responsible and intellectual working activities correlated with increased FC within the left IPL and the left cuneus. Our results are consistent with the actual literature on the functions of these areas and with other studies that reported the role of these regions in compensatory brain mechanisms [15, 59, 60].

The implication of the IPL in compensatory mechanisms was previously reported in patients with AD and aMCI [59, 60]. The IPL exhibited higher FC with the posterior regions of the DMN (e.g., posterior cingulate cortex and precuneus) in AD patients, which indicated that these patients may use additional different brain regions for information processing, probably as a compensation mechanism for cognitive decline [59]. Patterns of increased FC involving the IPL and areas of the DMN were also reported in aMCI patients and interpreted as the means of additional brain region recruitment needed to compensate for cognitive dysfunction [60]. Our results are consistent with the pivotal role of the IPL in diverse cognitive domains, such as visuospatial attention and stimulus-driven attention, which are essential in a constantly changing environment that demands that individuals adapt to behaviors quickly [61]. The IPL is tightly connected with many cortical areas and represents one of the key regions of the DMN [62, 63]. Consistent with our results of increased FC within the left IPL and the left cuneus, previous studies on healthy individuals reported a link between higher CR and higher FC of the cuneus in the DMN, which suggests a role of this region in the construction of tighter functional connections and higher network efficiency [4, 10]. A positive relationship between CR and the cuneus was reported in elderly individuals compared to younger individuals, which suggests that the higher the CR the more the elderly subjects engaged the cuneus [64]. A positive association between years of education and the nodal degree in the bilateral cuneus was also reported in patients with AD [15].

Overall, the present study demonstrated specific networks of increased internodal FC that positively correlated with increased levels of CR in SCA2 patients. Some limitations of the present study must be mentioned. One limitation is the fact that not all of the correlations survived after adjustment for multiple comparisons. Although Bonferroni is useful to avoid Type I error, it leads to a potentially dramatic increase in the number of Type II errors, namely the failure to detect differences that are actually significant [65]. Therefore, it was necessary to discuss the correlations that did not survive for multiple comparison adjustment. However, these results should be interpreted with caution. Another limitation is the small sample size of SCA2 patients. It has to be considered that the strict inclusion criteria and the fact that SCA2 is considered a rare disease with specific genetic roots clearly affected the recruitment rate. The limited number of patients do not permit a conclusive characterization of CR and associated rsfMRI connectivity patterns in patients with SCA2. This study is the first study to investigate CR and its substrate in SCA2 patients, and replications of these results on a larger group of patients are necessary. However,, considering the homogeneity of our sample and despite the small sample size, we reported consistent results that are consistent with the literature on CR in neurodegenerative diseases and suggest the importance of focusing on CR in SCA2 neurodegenerative disorder. Future studies are needed to confirm whether the network reorganization we hypothesized is also present in premorbid states of the disease. In addition, the assessment of CR longitudinally is required in order to verify the protective effect of CR on the long-term neuropsychological profile and connectivity patterns at rest of SCA2 patients. Hereof, it would be interesting to assess whether CR has positive impacts on cognitive functioning in every stage of the illness or whether this effect decreases longitudinally with increasing levels of neurodegeneration in each one patient.

Taken together, our results support the importance of identifying the status of CR that may relate to cognitive lifestyle activities in SCA2 patients and may be of clinical relevance for people who are at risk of developing the disease. In this regard, the early measurement of CR using precise tools, such as the CRIq, and the early identification of the genetic risk to develop the disease should be given further attention in the clinical setting with the aim of introducing timely and personalized clinical treatments for morbid and premorbid states to slow disease progression and improve the quality of life for patients and their families.

## Conclusions

In conclusion, the present study provided novel results on specific networks of increased internodal FC that positively correlated with increased levels of CR in SCA2 patients. In particular, the results suggest that specific functional networks involving regions that are highly susceptible to subserving neural reserve, such as vermis X, support network rearrangement expressly when SCA2 patients engaged in stimulating cognitive activities throughout the lifespan. Overall, we hypothesize that the increased FC involving cerebellar and cerebral nodes that correlated with CRIq indices may represent CR networks of neural reserve and compensatory mechanisms. This study shed light on the processes that allow SCA2 patients to cope with degenerative processes and be protected from cognitive decline.

### Supplementary Information

Below is the link to the electronic supplementary material.

Supplementary file1 (PDF 178 KB)

## Data Availability

The data that support the results of this study are available from the corresponding author upon reasonable request.
