# Supplementary material for: The rising role of cognitive reserve and associated compensatory brain networks in spinocerebellar ataxia type 2
Source: J Neurol. 2023 Jul 8;270(10):5071–84. doi: 10.1007/s00415-023-11855-3 (PMC10511586; doi:10.1007/s00415-023-11855-3)
Supplement: Supplementary file 1 — Supplementary file1 (PDF 178 KB) [file 415_2023_11855_MOESM1_ESM.pdf]

Table S1. Individual scores of International Cooperative Ataxia Rating Scale (ICARS) in SCA2 patients

ID	Posture and GaitDisturbances	Kinetic Functions	Speech Disorders	Oculomotor Disorders	ICARS TOT
**CB1**	13,5	28,5	3	2	47
**CB2**	12	11	2	1	26
**CB3**	13	13	2	0	28
**CB4**	13	10	3	1	27
**CB5**	13	13	3	2	31
**CB6**	11	15	2	0	28
**CB7**	18	15	3	3	39
**CB8**	7	7	2	1	17
**CB9**	8	11	3	2	24
**CB10**	12	13	3	1	29
**CB11**	11	12	0	1	24
**CB12**	24	30	5	2	61
**Means (SD)**	12,96 (4,44)	14,87 (7,06)	2,58 (1,16)	1,33 (0,89)	31,75 (11,95)

The global score of the ICARS ranges from 0 (absence of any motor deficit) to 100 (presence of motor deficits at the highest degree). The total score of the scale is determined by the sum of the subscores obtained in each section that evaluates specific ataxic symptoms: postural and gait disturbances (maximum score: 34), kinetic functions (maximum score: 52), speech disorders (maximum score: 8), oculomotor disorders (maximum score: 6).

Figure S1. Performances of SCA2 patients in cognitive domains expressed in Z-scores

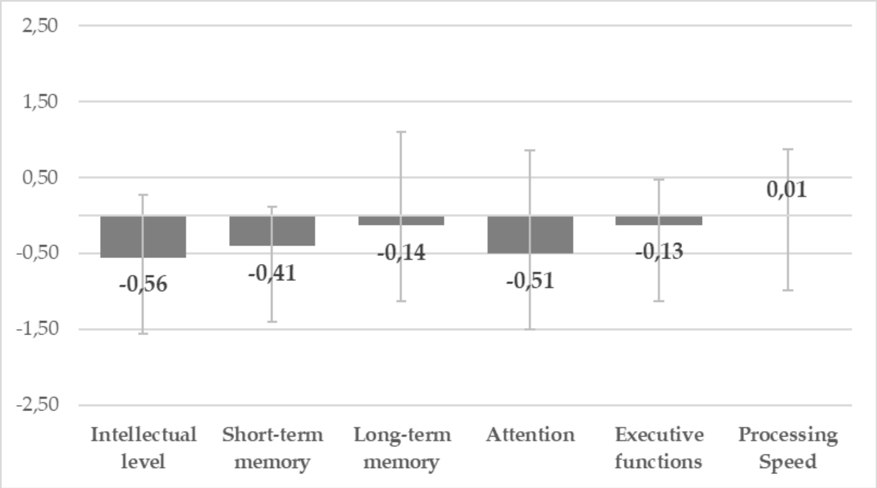

Mean and standard deviations of cognitive functions in SCA2 patients expressed in Z-scores (mean Z-scores are reported). Neuropsychological functions are grouped according to the cognitive domains assessed.

Table S2. Age, education and performances at the neuropsychological tests of the control groups.

Test		n°	Age mean /sd	Education mean /sd	Raw score mean /sd
Intellectual level	QI WAIS-r	32	42,42 /9,51	12,03/3,8	103,57 /10,76
Executive functions	Phonological fluency	72	48,14 /12,70	13,42 /3,66	40,77 /10,18
	WCST n° perseverative errors	43	47,44 /12,11	13,91 /3,48	7,95 /6,85

	Stroop Test - accuracy	43	47,44 /12,11	13,91 /3,48	0,31 /1,57
Shot-term memory	Rey's 15 words – Immediate recall *	340	53,1 /18,00	10,2 /4,30	42,31 /10,16
	Forward digit span	63	43,56 /14,58	12,17 /3,19	6,08 /1,24
	Backward digit span	63	43,56 /14,58	12,17 /3,19	4,45 /0,97
	Forward Corsi	63	51,13 /15,49	12,33 /4,12	5,82 /1,19
	Short-Story Recall - Immediate recall *	30	40 – 49	13,3 /3,4	5,95 /1,38
Long-term memory	Rey's 15 words – Delayed recall *	340	53,1 /18,00	10,2 /4,30	8,90 /3,16
	Short-Story Recall - Delayed recall *	30	40 – 49	13,3 /3,4	5,9 /1,45
Attention	MFTC - Accuracy *	465	62,1 /20,96	8,92 /5,32	0,94 /0,058
	Lines cancellation task – Accuracy *	40	69,3 /8,0	8,4 /4,2	59,80 /0,40
Processing speed	Stroop Test – execution time	43	47,44 /12,11	13,91 /3,48	19,17 /10,00
	MFTC - Execution time *	465	62,1 /20,96	8,92 /5,32	80,95 /40,07
	Lines cancellation task – Execution time *	40	69,3 /8,0	8,4 /4,2	47,30 /24,50

As reference means, normative data were used for the following tests: Rey's 15 Words, Short Story Test, MFTC, and the Line Cancellation Task. The population mean scores used as reference for the other tests were obtained from the scores of specific healthy control groups matched for mean age and education. * Published normative data; sd = standard deviation.

Table S3. Performances of the SCA2 patients at the neuropsychological tests.

	Test	Raw score mean /sd
Intellectual level	QI WAIS-r	89,7 /12,97
Executive functions	Phonological fluency	33,33 /10,62
	WCST n° perseverative errors	12,15 /10,76
	Stroop Test - accuracy	0,27 /0,52
Shot-term memory	Rey's 15 words – Immediate recall	44,07 /8,84
	Forward digit span	5,53 /0,66
	Backward digit span	4,07 /0,95
	Forward Corsi	5,25 /0,75
	Short-Story Recall - Immediate recall	4,53 /2,44
Long-term memory	Rey's 15 words – Delayed recall	10,38 /3,17
	Short-Story Recall - Delayed recall	5,17 /2,42
Attention	MFTC - Accuracy	0,93 /0,058
	Lines cancellation task – Accuracy	59,4 /1,07
Processing speed	Stroop Test – execution time	19,63 /7,77
	MFTC - Execution time	76,33 /34,86
	Lines cancellation task – Execution time	49,2 /26,80

sd = standard deviation.

Table S4. Correlations between Cognitive Reserve Index Questionnaire (CRIq) scores and patterns of internodal increased functional connectivity.

	CRI_Edu	CRI_WA	CRI_LA	CRI_Tot
R Frontal medial cortex ↔ L SMA	r = .073<br>p = .832	r = -.087<br>p = .800	r = .182<br>p = .592	r = .105<br>p = .759
Vermis X ↔ Lobules IV-V	r = .300<br>p = .370	r = .205<br>p = .545	***r = .606***<br>***p = .048***	r = .419<br>p = .199
R Frontal medial cortex ↔ R_SMA	r = -.064<br>p = .851	r = .092<br>p = .788	r = .253<br>p = .453	r = .147<br>p = .666
L_Cuneus ↔ L Inferior Parietal Lobule	r = .346<br>p = .297	***r = .685***<br>***p = .020***	r = .420<br>p = .198	***r = .653***<br>***p = .029***
R Frontal medial cortex ↔ R Temporal superior cortex	r = -.064<br>p = .852	r = .208<br>p = .540	r = .393<br>p = .232	r = .265<br>p = .431
L Parahippocampal Gyrus ↔ Vermis I-II	r = .382<br>p =.247	r =.442<br>p =.174	r = .328<br>p = .325	r =.465<br>p = .150
L Temporal medial cortex ↔ R Cerebellum X	r = -.144<br>p = .739	r = .023<br>p = .947	r = -.005<br>p = .989	r = -.046<br>p = .894
R Occipital medial cortex ↔ Vermis IV-V	r = .196<br>p = .564	r = -.087<br>p = .800	r = -.263<br>p = .435	r = -.201<br>p = .554
L Parahippocampal Gyrus ↔ Vermis X	***r = .881***<br>***p = .000***	r = .554<br>p = .077	r = .474<br>p = .141	***r = .783***<br>***p =.004***
R Fusiform Cortex ↔ Vermis_I-II	r = .036<br>p = .915	r = -.383<br>p = .245	r = -.469<br>p = .145	r = -.364<br>p = .270
L Fusiform Cortex ↔ Vermis X	r = .333<br>p = .318	r = .208<br>p = .540	r = .233<br>p = .491	r = .381<br>p = .247
R Fusiform Cortex ↔ Vermis X	***r = .843***<br>***p = .001***	r = .308<br>p = .356	r = .128<br>p = .708	r = .550<br>p = .079

Significant correlations ($p \leq .05$) are presented in bold italic type. CRIq_Edu = CRIq-Education; CRIq_WA = CRIq-WorkingActivity; CRIq_LA = CRIq-LeisureTime; R = right; L = left; SMA = Supplementar Motor Area.
